# Supplementary material for: First Isolation of a Herpesvirus (Family Alloherpesviridae) from Great Lakes Lake Sturgeon (Acipenser fulvescens)
Source: Animals (Basel). 2022 Nov 22;12(23):3230. doi: 10.3390/ani12233230 (PMC9740441; doi:10.3390/ani12233230)
Supplement: Supplementary file 1 [file animals-12-03230-s001.zip › animals-1994360-supplementary.pdf]

Table S1: The seventeen herpesviruses utilized for genomic comparisons and corresponding references.

Species	Virus	Host	Abbreviation	Strain	Accession Number	**Reference**
Ranid herpesvirus 1	Lucké tumor herpesvirus	*Rana pipiens*	RaHV1	McKinnell	DQ665917	50
Ranid herpesvirus 2	frog herpesvirus 4	*Rana pipiens*	RaHV2	Rafferty	DQ665652	51
Ranid herpesvirus 3	ranid herpesvirus 3	*Rana temporaria*	RaHV3	FO1_2015	KX832224	52
	bufonid herpesvirus 1	*Bufo bufo*	BfHV1	FO1_2015	MF143550	53
	ranid herpesvirus 4	*Hoplobatrachus rugulosus*	RaHV4	cxx6	MZ244210-MZ244215	54
Anguillid herpesvirus 1	Japanese eel herpesvirus	*Anguilla anguilla*	AngHV1	500138	FJ940765	55
Cyprinid herpesvirus 1	carp pox herpesvirus	*Cyprinus carpio*	CyHV1	NG-J1	JQ815363	36
Cyprinid herpesvirus 2	goldfish hematopoietic necrosis virus	*Carassius auratus*	CyHV2	ST-J1	JQ815364	36
Cyprinid herpesvirus 3	koi herpesvirus	*Cyprinus carpio*	CyHV3	KHV-U	DQ657948	56
Acipenserid herpesvirus 2	white sturgeon herpesvirus 2	*Acipenser transmontanus*	AciHV2	SRWSHV	FJ815289	57
Ictalurid herpesvirus 1	channel catifsh virus	*Ictalurus furcatus*	IcHV1	Auburn 1	M75136	58
Ictalurid herpesvirus 1	blue catfish alloherpesvirus	Ictalurus furcatus	IcHV1	S98-675	MK392382	59
Ictalurid herpesvirus 2	black bullhead herpesvirus	*Ameiurus melas*	IcHV2	760/94	MG271984	60
	silurid herpesvirus 1	*Kryptopterus bicirrhis*	SiHV1	KRB14001	MH048901	61
	acipenserid herpesvirus 1	*Acipenser transmontanus*	AciHV1	California	OK275723-OK275734	22
	lake sturgeon herpesvirus	*Acipenser fulvescens*	LSHV	Wolf River	OK485036	22
	sterlet herpesvirus	*Acipenser ruthenus*		Gen_M01	VTUV01000924	62

Table S2. Sequences producing significant (e-value < 1.0E-03) alignments (Blastn) with the 2,100 bp complete CDS of the Great Lakes lake sturgeon herpesvirus isolate 200413-11TC DNA packaging terminase subunit I.

Description	Acc. No.	% identity	alignment length (bp)	mismatches	e-value	bit score
Acipenserid herpesvirus 1 strain UC Davis	OK275734	85.7	2088	289	0	2422
Lake sturgeon herpesvirus strain Wolf River	OK485036	86.7	825	110	0	993
Lake sturgeon herpesvirus strain Wolf River	OK485036	93.3	570	38	0	857
Lake sturgeon herpesvirus strain Wolf River	OK485036	82.7	681	118	0	697
Acipenserid herpesvirus 1 isolate Italy	EF535575	90.7	290	27	2.00E-106	402
Acipenserid herpesvirus 1	EF535573	90.0	290	29	1.04E-103	393
Lake sturgeon herpesvirus isolate WVL19064-03	OL440177	99.4	156	1	5.74E-69	278
Lake sturgeon herpesvirus isolate WVL18106-01A	OL440176	99.4	156	1	5.74E-69	278
Lake sturgeon herpesvirus isolate WVL18090-01A	OL440175	99.4	156	1	5.74E-69	278
Lake sturgeon herpesvirus isolate WVL18089-02A	OL440173	99.4	156	1	5.74E-69	278
Lake sturgeon herpesvirus isolate WVL21072-4	OL440170	99.4	156	1	5.74E-69	278
Lake sturgeon herpesvirus isolate WVL17229-01A	OL440174	96.8	156	5	1.54E-63	260
Lake sturgeon herpesvirus isolate WVL19055-03	OL440172	96.2	156	6	1.88E-62	255
Lake sturgeon herpesvirus isolate WVL19077-02	OL440179	95.5	156	7	7.98E-61	251
Lake sturgeon herpesvirus isolate WVL19077-01	OL440178	95.5	156	7	7.98E-61	251
Lake sturgeon herpesvirus isolate WVL21072-1	OL440171	95.5	156	7	7.98E-61	251
Tiger shark herpes-like virus	MZ381454	65.9	369	115	2.01E-11	86
Ranid herpesvirus 2 strain ATCC VR-568	DQ665652	72.4	123	34	1.54E-06	69.8

Table S3. Sequences producing significant (e-value < 1.0E-03) alignments (Blastn) with the 4,638 bp complete CDS of Great Lakes lake sturgeon herpesvirus isolate 200413-11TC DNA polymerase catalytic subunit.

Description	Acc. No.	% identity	alignment (bp)	mismatches	e-value	bit score
Lake sturgeon herpesvirus strain Wolf River	OK485036	94.264	2859	143	0	4409
Lake sturgeon herpesvirus strain Wolf River	OK485036	90.777	1865	100	0	2631
Acipenserid herpesvirus 1 strain UC Davis	OK275729	80.449	4629	860	0	4250
Acipenserid herpesvirus 1 isolate WSHV-03-CA	EF685904	82.03	473	85	1.05E-126	471
Acipenserid herpesvirus 1 isolate AciHV1 DNA	EF685903	80.973	473	90	3.43E-120	448
Cyprinid herpesvirus 3 strain GY-01	MK260013	88.525	61	7	6.65E-09	79.7
Cyprinid herpesvirus 3 strain T	MG925491	88.525	61	7	6.65E-09	79.7
Cyprinid herpesvirus 3 strain M3	MG925490	88.525	61	7	6.65E-09	79.7
Cyprinid herpesvirus 3 strain I	MG925489	88.525	61	7	6.65E-09	79.7
Cyprinid herpesvirus 3 strain GZ11-SC	MG925488	88.525	61	7	6.65E-09	79.7
Cyprinid herpesvirus 3 strain FL	MG925487	88.525	61	7	6.65E-09	79.7
Cyprinid herpesvirus 3 strain E	MG925486	88.525	61	7	6.65E-09	79.7
Cyprinid herpesvirus 3 strain Cavoy	MG925485	88.525	61	7	6.65E-09	79.7
Cyprinid herpesvirus 3 isolate PP3_070411	KX544848	88.525	61	7	6.65E-09	79.7
Cyprinid herpesvirus 3 isolate I_10_3	KX544847	88.525	61	7	6.65E-09	79.7
Cyprinid herpesvirus 3 isolate I_09_2i3	KX544846	88.525	61	7	6.65E-09	79.7
Cyprinid herpesvirus 3 isolate J1_101110	KX544845	88.525	61	7	6.65E-09	79.7
Cyprinid herpesvirus 3 isolate CB4_181110	KX544844	88.525	61	7	6.65E-09	79.7
Cyprinid herpesvirus 3 isolate J2_101110	KX544843	88.525	61	7	6.65E-09	79.7
Cyprinid herpesvirus 3 isolate PoB3	KX544842	88.525	61	7	6.65E-09	79.7

Cyprinid herpesvirus 3 isolate FL BAC revertant ORF56-57	KP343684	88.525	61	7	6.65E-09	79.7
Cyprinid herpesvirus 3 isolate FL BAC revertant ORF136	KP343683	88.525	61	7	6.65E-09	79.7
Cyprinid herpesvirus 3 strain KHV-GZ11	KJ627438	88.525	61	7	6.65E-09	79.7
Cyprinid herpesvirus 3 isolate MN	MT914509	88.525	61	7	6.65E-09	79.7
Cyprinid herpesvirus 3 DNA TUMST1	AP008984	88.525	61	7	6.65E-09	79.7
Cyprinid herpesvirus 3 strain KHV-U	DQ657948	88.525	61	7	6.65E-09	79.7
Koi herpesvirus strain KHV-I	DQ177346	88.525	61	7	6.65E-09	79.7
Koi herpesvirus isolate 323909	DQ128163	88.525	61	7	6.65E-09	79.7
Cyprinid herpesvirus 3 KHV-JG01	AB196133	88.525	61	7	6.65E-09	79.7
Cyprinid herpesvirus 3 KHV-JG07	AB196131	88.525	61	7	6.65E-09	79.7
Cyprinid herpesvirus 3 KHV-JG05	AB195962	88.525	61	7	6.65E-09	79.7
Cyprinid herpesvirus 3 KHV NRIA 0301	AB195961	88.525	61	7	6.65E-09	79.7
Cyprinid herpesvirus 3 KHV-I	AB195960	88.525	61	7	6.65E-09	79.7
Cyprinid herpesvirus 3 KHV-U	AB195959	88.525	61	7	6.65E-09	79.7
Cyprinid herpesvirus 1 isolate Ma1	MK507842	83.333	72	12	2.32E-08	77
Cyprinid herpesvirus 1 strain NG-J1	JQ815363	83.333	72	12	2.32E-08	77
Anguillid herpesvirus 1 isolate HVA980811	KX027736	86.792	53	7	1.47E-04	65.3
Anguillid herpesvirus 1 strain UK N080	MW580855	86.792	53	7	1.47E-04	65.3
Anguillid herpesvirus 1 strain HVA 486123	MW580854	86.792	53	7	1.47E-04	65.3
Anguillid herpesvirus 1 strain DK-206116-1	MW580853	86.792	53	7	1.47E-04	65.3
Anguillid herpesvirus 1 strain DK-205223-2	MW580852	86.792	53	7	1.47E-04	65.3

Anguillid herpesvirus 1 strain DK-200249	MW580851	86.792	53	7	1.47E-04	65.3
Anguillid herpesvirus 1 strain DK-2008-50-66-1	MW580850	86.792	53	7	1.47E-04	65.3
Anguillid herpesvirus 1 strain 500138	MW580849	86.792	53	7	1.47E-04	65.3
Anguillid herpesvirus 1 isolate C3P2	GU233800	86.792	53	7	1.47E-04	65.3

Table S4. Average nucleotide identity estimations for channel catfish virus (IcHV1; NC_001493) and blue catfish alloherpesvirus (IcHV1; MK392382).

CCV vs BCAHV				
Minimum alignment	Minimum identity	One-way ANI 1	One-way ANI 2	Two-way ANI
125	70%	93.38% (SD: 4.79%), from 651 fragments.	93.42% (SD: 4.75%), from 651 fragments.	94.73% (SD: 3.49%), from 525 fragments.
125	75%	93.38% (SD: 4.79%), from 651 fragments.	93.42% (SD: 4.75%), from 651 fragments.	94.73% (SD: 3.49%), from 525 fragments.
125	80%	93.48% (SD: 4.59%), from 649 fragments.	93.52% (SD: 4.53%), from 648 fragments.	94.73% (SD: 3.49%), from 525 fragments.
125	85%	93.87% (SD: 3.96%), from 635 fragments.	93.90% (SD: 3.92%), from 636 fragments.	94.78% (SD: 3.37%), from 524 fragments.
250	70%	93.38% (SD: 4.79%), from 651 fragments.	93.41% (SD: 4.79%), from 650 fragments.	94.73% (SD: 3.49%), from 525 fragments.
250	75%	93.38% (SD: 4.79%), from 651 fragments.	93.41% (SD: 4.79%), from 650 fragments.	94.73% (SD: 3.49%), from 525 fragments.
250	80%	93.49% (SD: 4.59%), from 647 fragments.	93.51% (SD: 4.57%), from 647 fragments.	94.73% (SD: 3.49%), from 525 fragments.
250	85%	93.91% (SD: 3.96%), from 628 fragments.	93.96% (SD: 3.92%), from 627 fragments.	94.79% (SD: 3.37%), from 523 fragments.
500	70%	93.71% (SD: 4.52%), from 620 fragments.	93.69% (SD: 4.59%), from 623 fragments.	94.73% (SD: 3.49%), from 525 fragments.
500	75%	93.71% (SD: 4.52%), from 620 fragments.	93.69% (SD: 4.59%), from 623 fragments.	94.73% (SD: 3.49%), from 525 fragments.
500	80%	93.80% (SD: 4.34%), from 617 fragments.	93.82% (SD: 4.33%), from 619 fragments.	94.73% (SD: 3.49%), from 525 fragments.
500	85%	94.13% (SD: 3.82%), from 603 fragments.	94.14% (SD: 3.82%), from 605 fragments.	94.79% (SD: 3.37%), from 523 fragments.
750	70%	94.08% (SD: 4.16%), from 586 fragments.	94.12% (SD: 4.16%), from 584 fragments.	94.76% (SD: 3.46%), from 523 fragments.
750	75%	94.08% (SD: 4.16%), from 586 fragments.	94.12% (SD: 4.16%), from 584 fragments.	94.76% (SD: 3.46%), from 523 fragments.
750	80%	94.12% (SD: 4.09%), from 585 fragments.	94.19% (SD: 4.02%), from 582 fragments.	94.76% (SD: 3.46%), from 523 fragments.
750	85%	94.35% (SD: 3.67%), from 576 fragments.	94.39% (SD: 3.66%), from 574 fragments.	94.82% (SD: 3.34%), from 521 fragments.

Table S5. Average nucleotide identity estimations for Cyprinid herpesvirus 1 (CyHV1; JQ815363) and Cyprinid herpesvirus 3 (CyHV3; MK260013).

CyHV1 vs CyHV3				
Minimum alignment		Minimum identity	One-way ANI 1	One-way ANI 2
125	70%	74.15% (SD: 4.61%), from 78 fragments.	74.46% (SD: 4.43%), from 76 fragments.	Insufficient hits
125	75%	75.97% (SD: 3.86%), from 58 fragments.	76.42% (SD: 3.56%), from 55 fragments.	Insufficient hits
125	80%	Insufficient hits	Insufficient hits	Insufficient hits
125	85%	Insufficient hits	Insufficient hits	Insufficient hits
250	70%	72.90% (SD: 3.98%), from 57 fragments.	73.33% (SD: 4.02%), from 54 fragments.	Insufficient hits
250	75%	Insufficient hits	Insufficient hits	Insufficient hits
250	80%	Not tested	Not tested	Not tested
250	85%	Not tested	Not tested	Not tested
500	70%	Insufficient hits	Insufficient hits	Insufficient hits
500	75%	Insufficient hits	Insufficient hits	Insufficient hits
500	80%	Not tested	Not tested	Not tested
500	85%	Not tested	Not tested	Not tested
750	70%	Insufficient hits	Insufficient hits	Insufficient hits
750	75%	Not tested	Not tested	Not tested
750	80%	Not tested	Not tested	Not tested
750	85%	Not tested	Not tested	Not tested
